# Integrative proteogenomic analyses of human tumours identifies ADNP as a novel oncogenic mediator of cell cycle progression in high-grade serous ovarian cancer with poor prognosis

**DOI:** 10.1016/j.ebiom.2019.11.009

**Published:** 2019-11-22

**Authors:** Kubra Karagoz, Gaurav A. Mehta, Christen A. Khella, Pooja Khanna, Michael L. Gatza

**Affiliations:** a Rutgers Cancer Institute of New Jersey, New Brunswick, NJ, United States; b Department of Radiation Oncology, Robert Wood Johnson Medical School, United States; c Rutgers, The State University of New Jersey, New Brunswick NJ, United States

**Keywords:** High-grade serous ovarian cancer, HGSOC, Proteogenomic, ADNP, Poor prognostic marker

## Abstract

**Background:**

Despite toxic side effects and limited durable response, the current standard-of-care treatment for high grade serous ovarian cancer (HGSOC) remains platinum/taxane-based chemotherapy. Given that the overall prognosis has not improved drastically over the past several decades, there is a critical need to understand the underlying mechanisms that lead to tumour development and progression.

**Methods:**

We utilized an integrative proteogenomic analysis of HGSOC tumours applying a poor prognosis gene expression signature (PPS) as a conceptual framework to analyse orthogonal genomic and proteomic data from the TCGA (*n* = 488) and CPTAC (*n* = 169) studies. Genes identified through *in silico* analyses were assessed *in vitro* studies to demonstrate their impact on proliferation and cell cycle progression.

**Findings:**

These analyses identified DNA amplification and overexpression of the transcription factor *ADNP* (Activity Dependent Neuroprotector Homeobox) in poorly prognostic tumours. Validation studies confirmed the prognostic capacity of *ADNP* and suggested an oncogenic role for this protein given the association between *ADNP* expression and pro-proliferative signalling. *In vitro* studies confirmed *ADNP* as a novel and essential mediator of cell proliferation through dysregulation of cell cycle checkpoints.

**Interpretation:**

We identified *ADNP* as being amplified and overexpressed in poor prognosis HGSOC *in silico* analyses and demonstrated that *ADNP* is a novel and essential oncogene in HGSOC which mediates proliferation through dysregulation of cell cycle checkpoints *in vitro*.

**Funding:**

The National Cancer Institute of the National Institutes of Health, the V Foundation for Cancer Research and the New Jersey Commission for Cancer Research.

**Research in context**

**Evidence before this study**

Ovarian cancer is the fifth-leading cause of cancer death among women in the United States. Despite extensive multi-omics characterization of high grade serous ovarian cancer (HGSOC) and improved treatment strategies, the overall 5-year survival rate continues to trail most other malignancies. Thus, it is urgent to identify novel therapeutic targets and biomarkers.

**Added value of this study**

In this study, integrative proteogenomic analyses of HGSOC tumours identified *ADNP* as a potential novel driver of HGSOC. We confirmed the prognostic capacity of *ADNP* in multiple independent datasets and *in vitro* studies showed the essentiality of this protein in regulating cell proliferation and survival. Our analyses demonstrate that *ADNP* regulates HGSOC tumorigenesis by promoting dysregulation of cell cycle checkpoints.

**Implications of all the available evidence**

Our findings indicated that *ADNP* is poor prognostic marker in multiple datasets. Importantly, we validated that *ADNP* mediates cell proliferation through dysregulation of cell cycle checkpoints in ovarian cancer. Our findings supported *ADNP* as a novel oncogenic driver of HGSOC growth and survival.

Alt-text: Unlabelled box

## 1 Introduction

Ovarian cancer is the fifth leading cause of cancer-related deaths among women in the United States in 2019 [1]. The most common histological subtype of epithelial ovarian cancer is high-grade serous ovarian cancer (HGSOC). Although most patients initially respond to platinum–taxane based chemotherapy and surgical resection, most tumours recur and become increasingly resistant to chemotherapy [2].

HGSOC tumours express a relatively homogenous somatic or germline mutation profile and are characterized by *TP53* mutations in >90% of tumours as well as frequent *BRCA1* and *BRCA2* mutations [3]. Although these mutations occur at a high frequency, HGSOC tumors have been shown to be C class tumors characterized by recurrent DNA copy number alterations and few other common mutations. [4]. As was shown by the Cancer Genome Atlas (TCGA) project [3], these alterations manifest as dysregulated Rb/E2F, Ras/PI3K, FoxM1 and Notch signalling; however clinical trials have generally demonstrated a lack of response in these tumours to inhibition of these pathways [5,6]. A number of previous studies, including those from the TCGA and Clinical Proteomic Tumour Analysis Consortium (CPTAC) projects have demonstrated that HGSOC can be classified into multiple transcriptome or proteome-based classes [3,7,8]. While these subtypes do exhibit unique genomic and/or proteomic patterns, the prognostic capacity of these groups remains unclear as several conflicting studies have been reported [3]. While the TCGA initially demonstrated no significant prognostic difference between these groups, more recent studies have suggested that the proliferative and mesenchymal subtypes may have a worse prognosis when compared to the immunoreactive subtype [9,10]. Interestingly, a recent study has suggested that these subtypes may benefit from addition of bevacizumab [9]. Regardless, the general lack of drug-able targets expressed in HGSOC tumours and the reality that the overall prognosis for HGSOC has not improved drastically over the past several decades, despite the recent inclusion of PARP inhibitors [11], suggests that there is a critical need to understand the mechanisms that lead to tumour development and progression.

To identify genes responsible for regulating specific signalling pathways and/or tumorigenic properties that contribute to poor clinical outcome, we utilized a previously published Poor Prognosis Signature (PPS) [3] as a conceptual framework to perform integrative proteogenomic analyses of human HGSOC tumours. Our analyses identified increased DNA copy number gains and higher mRNA and protein expression of the transcription factor *ADNP* (Activity Dependent Neuroprotector Homeobox) in poorly prognostic HGSOC tumours.

*ADNP* is a Homeobox transcription regulator which includes nine zinc-fingers that plays a role in neuroprotective responses to cellular growth, chromatin remodelling, microtubule/autophagy regulation and cell proliferation [12], [13], [14], [15] While *ADNP* is localized to 20q12, a chromosomal region that is frequently amplified and/or overexpressed in many human malignancies including HGSOC, breast, pancreatic, and colon cancers [16], the majority of published studies have focused on the role of *ADNP* in neurological development and disease including autism spectrum disorders and Alzheimer's disease [17]. As a result, the role of *ADNP* in cancer, or HGSOC specifically, has not been extensively studied. Aberrant expression of *ADNP* has been reported to mediate intestinal cell growth, proliferation in specific types of sarcomas and neuronal tissue and to modulate expression of E2F-regulated genes as well as PI3K/ AKT signalling [15,[18], [19], [20]] suggesting that it may play an oncogenic role in specific tumour types or cellular environments. Consistent with this premise, our analyses determined that *ADNP* expression is prognostic in multiple HGSOC datasets and *in vitro* studies demonstrate the *ADNP* mediates the expression of key cell cycle genes and is required for HGSOC cell growth and survival thereby supporting a role for *ADNP* in HGSOC tumorigenesis.

## 2 Materials and methods

### 2.1 Human tumour and cell line multi-omics data

Affymetrix HT-HG-U133A microarray data for HGSOC samples was obtained from the Firehose data portal (https://gdac.broadinstitute.org/). Affymetrix U133Plus2.0 microarray data for the 285 patients Tothill (GSE9891) [7] and 260 patients Yoshihara [21] (GSE32062) datasets as well as 29 ovarian cancer cell lines (GSE36139) [22] were acquired from the Gene Expression Omnibus (GEO). RNASeq data of HCT116 colorectal cancer cells treated with siControl or siADNP (*n* = 6) were obtained from GEO (GSE79395) [23].

GISTIC 2.0 [24] segmentation scores as well as threshold copy number calls (*i.e.* −2, −1, 0, 1, or 2) for the 488 TCGA samples with corresponding mRNA expression data were acquired (April 2015) from the Firehose data portal. Reverse Phase Protein Array (RPPA) data for 190 proteins and phosphoproteins from 338 HGSOC samples in the TCGA cohort were acquired (June 2015) from the Firehose data portal. In addition, we acquired mass spectrometry data (*n* = 3586 proteins) for 169 samples from the CPTAC study [8].

### 2.2 Gene expression signatures

A panel of 62 previously published gene expression signatures was used to examine patterns of pathway activity and/or microenvironmental states (Table S1). To implement each signature, the methods detailed in the original studies were followed as closely as possible. The list of signatures is shown in Table S1 and the scores for the TCGA data set (Table S2), Tothill (Table S3) and Yoshihira (Table S4) are reported.

### 2.3 Statistical analyses of signature scores

**Fig. 1:**
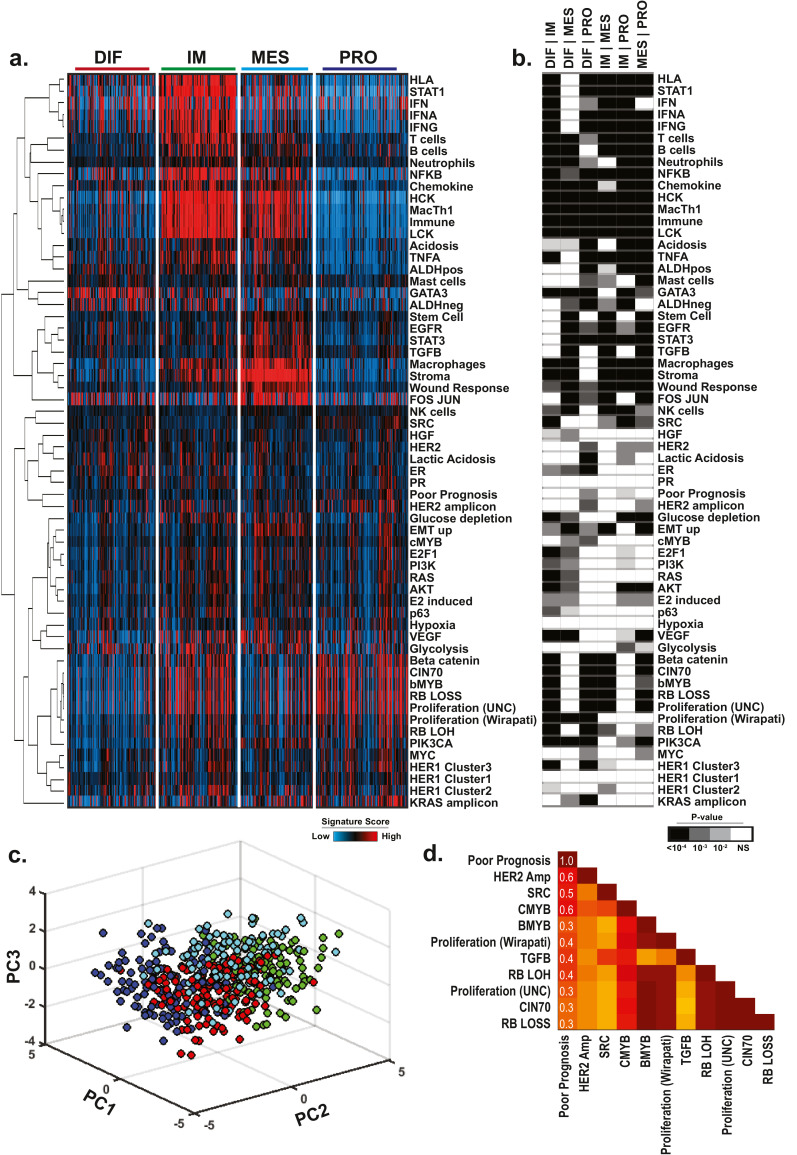
Patterns of pathway activity in HGSOC. (a) Patterns of pathway activity (*n* = 62) were determined for each sample (*n* = 488) in the TCGA high-grade serous ovarian cancer cohort and plotted relative to the previously described molecular subtypes. Expression signatures (*y*-axis) are median centered and clustered by complete linkage hierarchical clustering (b) ANOVA test followed by Tukey pairwise comparison was used to demonstrate statistically significant difference in signature score across the molecular subtypes. (c) Principal Component Analysis (PCA) was used to demonstrate the spatial distribution of subtypes based on the expression patterns of the 62 signature scores. Subtype colouring is the same as in (a). (d) Pearson Correlation coefficient (r-values) for the top 10 most strongly and consistently correlated pathways are shown relative to the Poor Prognosis Signature (PPS) for the TCGA cohort.

To quantify differences in patterns of signature scores across subtypes, a two-way ANOVA followed by Tukey's post-test for pairwise comparisons was used (Fig. 1b and Table S5). Principal component analysis (PCA) was performed to assess the distribution of TCGA tumour samples across transcriptomic subtypes [3] using pathway activity scores. A Pearson correlation was used to determine the correlation coefficient (*R*-value) for each pairwise relationship between each signature and the Poor Prognosis Signature (PPS) for samples in the TGCA (Table S6), Tothill (Table S7) and Yoshihara (Table S8) studies. The top 10 most consistently positively correlated signatures associated with the PPS score are reported in Fig. 1 and Fig. S1.

### 2.4 Identification of genomic and proteomic alterations associated with poor prognosis

Pathway–specific copy number alterations (CNA), were identified using a previously described strategy [25,26] by using a Spearman rank correlation and Fisher's exact test. For each analysis, the Benjamin-Hochberg (BH) adjusted *P* values are reported and a threshold of *P* < 0.05 (BH corrected) was set (Table S9). A Spearman rank correlation was used to evaluate the association between poor prognosis and mRNA and protein; these data are reported in Table S10-11.

### 2.5 Analysis of genome-wide RNAi proliferation screen data

To assess the essentiality of *ADNP* relative to the PPS signature score, ovarian cancer cell line shRNA abundance data was acquired from the Broad Institute Project Achilles dataset [27]. These data were filtered to include only those 29 cell lines for which gene expression data (GSE36139) were also available [22]. A Spearman rank correlation was used to calculate the negative correlation between *ADNP* shRNA abundance and PPS score.

### 2.6 Survival analyses

To confirm the prognostic capacity of the PPS signature, samples from the TCGA (*n* = 564), Tothill (*n* = 256) and Yoshihara (*n* = 260) studies were assessed. Clinical data from each study was acquired as detailed above. Overall survival (OS) in each dataset was assessed by comparing patients with high (PPS^High^; top quartile) and low PPS score (PPS^Low^; bottom quartile) by log-rank test; *p*-value and hazard ratio are reported in Fig. S1. To demonstrate the relationship between *ADNP* copy number status or mRNA expression level and overall survival (OS), three datasets were assessed. For copy number-based analyses, OS was determined for TCGA samples with a low level copy number gain or amplification as compared to all other samples (*i.e.* diploid, LOH or deletion). To assess difference in OS based on *ADNP* mRNA level, samples were dichotomized into ADNP^High^ (top quartile) and ADNP^Low^ (bottom quartile) in the Tothill and Yoshihara datasets. For each analysis, significance was calculated by a log-rank test and the hazard ratio (HR) is reported.

### 2.7 Cell culture and shRNA knockdown

Ovarian cancer cell lines were purchased from the American Tissue Culture Collection (Manassas, VA, USA) and cultured according to the suggested guidelines. OVCAR3 or OVCAR5 cell lines expressing one of two tetracycline (tet)-inducible shRNA expressing cell lines were created using the pTRIPZ Inducible Lentiviral shRNA system (GE Dharmacon). The catalogue number for shRNA(1) is: V3THS_313782 and for shRNA(2) is: V3THS_313783. The shRNA expression was induced using 1.0 µg/mL of doxycycline and *ADNP* silencing was verified by qRT-PCR and/or western blot analyses.

### 2.8 Western blot analysis

50 µg of protein was loaded on 4–20% Mini-protean TGX gradient gel (BioRad). Proteins were separated at 100 V for 2 h at room temperature and then transferred onto nitrocellulose membrane at 100 V for 1 h at 4 °C. The membranes were blocked using 5% milk solution, incubated with primary antibody against total ADNP or beta-actin (Cell Signalling Technology) overnight at 4 °C followed by incubation with HRP-conjugated secondary antibodies (Cell Signalling Technology) for 1 h at room temperature. The signal was developed using SuperSignal West Pico Chemiluminescent Substrate (ThermoFisher Scientific), digitally imaged using the ChemiDoc Touch Imagining System (BioRad).

### 2.9 Quantitative real-time PCR

Total RNA was isolated using RNeasy plus Mini Kit (Qiagen) and cDNA was synthesized using the QuantiTect Reverse Transcription kit (Qiagen). Quantitative PCR (qPCR) was performed and analysed using the Applied Biosystems QuantStudio3 real time thermal cycler system. Primer utilized for human genes are as follow ADNP Forward: 5′-GGATTTTGGCGTCTTCTCAG-3′, *ADNP* Reverse: 5′-AGC GGTGCAGACAAAGGA-3′, GAPDH Forward: 5′-TCTGACTTCAACAGCGACAC-3′, *GAPDH* Reverse: 5′-CCAGCCACATACCAGGAAAT-3′, *CDC25A* Forward: 5′-GAGGAGTCTCACCTGGAAGTACA-3′, *CDC25A* Reverse: 5′-GCCATTCAA AACCAGATGCCATAA-3′, *WEE1* Forward: 5′- GCGTGGTAGCACACATCATT-3′, *WEE1* Reverse: 5′-GTGCAATCACGGCTCTGTAG-3′, *CCNB1* Forward: 5′- ATGACATGGTGCACTTTCCTCC-3′, *CCNB1* Reverse: 5′-GCCAGGTGCTGCATAACTGG-3′, *CCNB2* Forward: 5′-GATAACGAAGATTGGGAGAACCC-3′, *CCNB2* Reverse: 5′-CCACTAGGATGGCACGCATG-3′, *CCNE1* Forward: 5′-CAAACTCAACGTGCAAGCCTC-3′, *CCNE1* Reverse: 5′-GCCCAGCTCAGTACAGGCAG-3′, *CCNE2* Forward: 5′-AATTACATAAACACCTTCAGAAAAGGG3′, and *CCNE2* Reverse: 5′-GTGCTCTTCGGTGGTGTCATAA-3′.

### 2.10 Cell proliferation assay and colony formation assay

For cell proliferation assay, OVCAR3 and OVCAR5 cells were mock treated or treated with doxycycline (1 µg/mL) for 96 h. Cell proliferation was assessed by the CellTiter 96 AQueous One Cell proliferation assay (Promega BioSciences) according to the manufacture's protocol. For colony formation assay, OVCAR3 and OVCAR5 cells were mock treated or treated with doxycycline (1 µg/mL) and grown for 14 days for OVCAR3 or 7 days for OVCAR5. The cells were stained with 0.2% crystal violet in 95% ethanol and photographed; colonies were manually quantified for each experimental replicate and normalized to the untreated control.

### 2.11 Cell cycle assay

OVCAR3 and OVCAR5 cells were treated either mock or doxycycline treated for 96 h. DNA content was assessed by Sytox Green staining (50 *μ*g/mL) for 30 min in the dark. The cell cycle distribution was analysed by Beckman-Coulter Cytomics FC500 Flow Cytometer. The percentage of cells in G0/G1 and G2/M were determined for a minimum of 3 independent experiments.

### 2.12 Apoptosis assay

OVCAR3 and OVCAR5 cells were either mock treated or treated with doxycycline for 96 h before apoptosis assays. After 96 h of treatment, the percentage of apoptotic cells were determined by Annexin V-FITC/Hoechst 33342 staining using the Dead Cell Apoptosis Kit with Annexin V-FITC and Hoechst 33342 (Invitrogen) according to the manufacturer's instructions.

## 3 Results

### 3.1 Identification of subtype-specific patterns of oncogenic activity

In order to identify genetic drivers of HGSOC that contribute to tumour aggressiveness and are associated with poor overall survival, we first examined patterns of oncogenic signalling, the tumour microenvironment, immune infiltration, and other essential tumorigenic characteristics in human ovarian tumours. A panel of 62 previously published gene expression signatures (Tables S1 and S2), including a Poor Prognosis Signature (PPS) [3], was applied to HGSOC gene expression microarray data (*n* = 488) from the TCGA study (Table S3) for which the molecular subtype had been determined [3] (Fig. 1a). While the PPS signature was only modestly enriched in the proliferative subgroup, patterns of pathway activity were able to recapitulate some known features of each molecular subtype. For instance, immune signatures including T cells [28], B cells [28]), macrophage [28], HCK [29], LCK [29], and other immune-related signatures were shown to be consistently up-regulated in the immunoreactive (IR) subtype (Fig. 1a). Likewise, signatures associated with epithelial-to-mesenchymal transition including the cancer stem cell [30], stroma-associated signalling [31]) and TGFβ [25,32] signatures were found to be activated in the mesenchymal subtype. Finally, several proliferation-associated signatures were found to be modestly up-regulated in the proliferative subtype.

In contrast to previous studies in breast cancer [25] which showed robust and uniform subtype-specific patterns of pathway activity, with the above noted exceptions, consistent patterns of oncogenic pathway activity, including MYC, RAS, AKT and RB/E2F1 were not observed across HGSOC subtypes. This observation is more readily apparent when differences in patterns of pathway activity are quantitatively assessed by an analysis of variance (ANOVA) test followed by Tukey's test for pairwise comparison (Fig. 1b and Table S4). Further analysis of subtype-specific patterns of pathway activity by Principal Component Analysis (PCA) indicated that while molecular subtypes could be distinguished amongst the entire dataset based on pathway patterns, HGSOC tumours appear to be largely homogeneous in nature with respect to these specific molecular characteristics (Fig. 1c). This observation is consistent with previous studies indicating a higher degree of homogeneity between HGSOC tumours relative to the diversity observed in other tumour types [3].

Given the lack of subtype-specific oncogenic signalling, we next sought to identify oncogenic signalling or other tumour features associated with prognosis. To do so, we took advantage of the previously published Poor Prognosis Signature (PPS) [3]. We first validated the prognostic capacity of this signature in three independent datasets. As illustrated in Fig S1, the PPS signature was applied to the TCGA (Fig. S1a), Tothill [7] (Fig. S1b) and Yoshihara [21] (Fig. S1c) datasets. Patients were then dichotomized into PPS^High^ (top quartile) and PPS^Low^ (bottom quartile) subgroups (Fig. S1d–f) to examine differences in overall survival. As expected, the PPS^High^ subgroup of patients consistently showed an overall worse prognosis (Fig. S1g–i) thereby validating the prognostic capacity of the PPS signature.

A Pearson Correlation was next used to calculate the concordance between the PPS signature and all other signatures in the TCGA, Tothill [7] and Yoshihara [21] datasets (Tables S5-S6). As illustrated in Fig. 1d (and Fig. S1j–l) the top 10 most consistent and strongly concordant signalling pathways across each dataset associated with the PPS signature were identified. These analyses demonstrated that poor prognosis may be associated with altered cell cycle progression, chromosome instability and proliferation as illustrated by the observed correlation between PPS signature and RB LOH [33], RB Loss [34], CMYB [35], bMYB [36], and Chromosome Instability 70 gene signature (CIN70) [37] signatures as well as two independent proliferation signatures [38,39]. Although these associations are statistically significant (*p* < 0.05), modest correlation coefficients (*r* = 0.1 to 0.6) suggest that other genomic events or altered genes not directly identified by these analyses may also contribute to HGSOC development and poor prognosis.

### 3.2 Identification of genomic and proteomic alterations associated with PPS activity

**Fig. 2:**
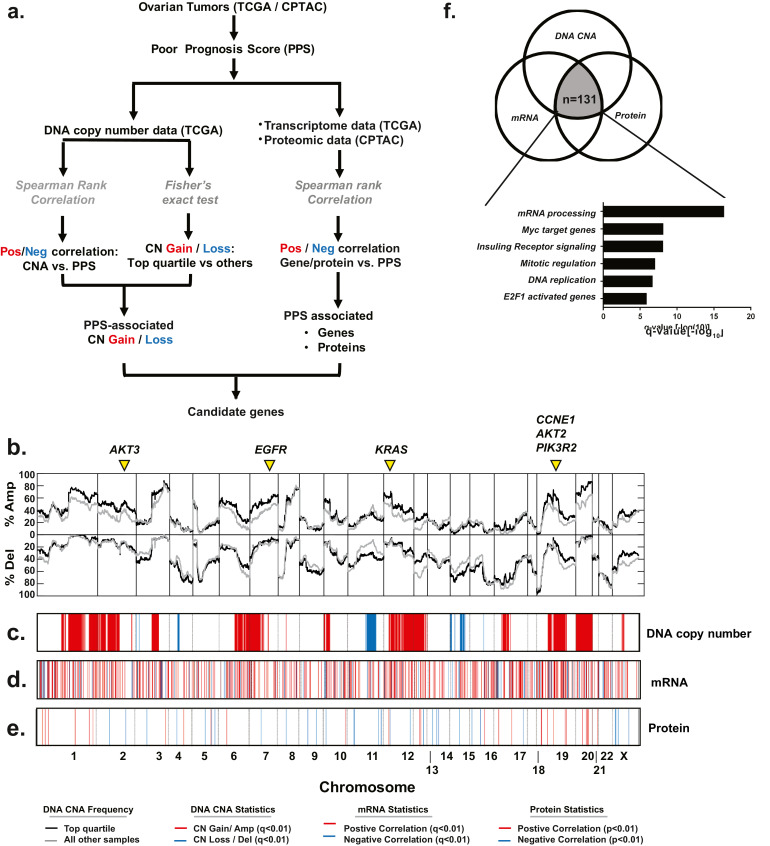
Identification of altered genes and proteins associated with PPS activity. (a) Schematic outlining the strategy to identify copy number alterations, transcript and proteins that correspond to PPS activity in the TCGA and CPTAC datasets. (b) Identification of statistically associated copy number alterations.The plot demonstrates the frequency of copy number gains/amplification events (above the horizontal axis) or deletions/ LOH (below the horizontal axis) for each gene in the PPS^High^ (top quartile; black line) or PPS^Low^ (all other quartiles; grey line) cohorts; a Fisher's exact test was used to calculate statistical significance. (c) Plot identifies genes that have a statistically significant correlation between DNA copy number gain (red) or loss (blue) relative to the PPS score as determined by an FDR-corrected Spearman rank correlation (Benjamini-Hockberg). (d) Identification of genes that demonstrate a positive (red) or negative (blue) association between mRNA expression and PPS score using an FDR-corrected Spearman rank correlation (Benjamini-Hockberg). (e) An FDR-corrected Spearman rank correlation (Benjamini-Hockberg) was used to identify proteins that demonstrate a positive (red) or negative (blue) association with PPS score. (f) Venn diagram identifying 131 genes that have a copy number gain/amplification in PPS^High^ samples and showed a positive mRNA and protein correlation with PPS activity; GSEA analysis identified six predominant activities affected by these genes. (For interpretation of the references to color in this figure legend, the reader is referred to the web version of this article.)

We next identified genomic and proteomic alterations directly associated with poor prognosis in HGSOC, including potential novel drivers of HGSOC oncogenesis. To do so, we utilized an integrated proteogenomics strategy based on the use of the previously discussed PPS signature as a conceptual framework to interrogate orthogonal genomic and proteomic data from the TCGA [3] (*n* = 488) and CPTAC [8] (*n* = 169) studies. We have outlined the scheme used for our analysis to identify DNA copy number alterations as well as altered mRNA expression and protein expression associated with the PPS signature in Fig. 2a.

Given the high level of chromosomal instability in HGSOC, we first identified DNA copy number alterations that were unique to tumours with a high PPS score using a previously published approach [25]. To identify PPS-specific copy number gains or losses, we used two approaches to minimize potential biases that might be associated with either strategy alone. First, using DNA copy number (CN) data from TCGA samples (*n* = 488), we identified those genes that showed an increased in CN gains (either a low level gain or high-level amplification) or losses (either loss of heterozygosity or deletion) in the PPS^High^ (top quartile) tumours compared to PPS^Low^ (all other samples) tumours using an FDR-corrected (Benjamini–Hockberg) Fisher's Exact test (Fig. 2b). To confirm the association between CN frequency and PPS score, an FDR-corrected Spearman rank correlation (Benjamini–Hockberg) between DNA segment score and PPS score was used as a secondary analysis. Genes that demonstrated an increase in CN gains in PPS^High^ samples and had a positive Spearman rank correlation were considered PPS-specific gains whereas those genes that showed increased CN losses in PPS^High^ samples and a negative correlation were considered PPS-specific losses. Requiring that PPS-specific copy number alterations meet each of these criterion (*q* < 0.05) reduces potential false-positives associated with either strategy alone; chromosomal regions that met these thresholds are illustrated in Fig. 2c and summarized in Table S7.

Consistent with previous studies reporting that HGSOC tumours often exhibited alterations in one or more components of the RAS/PI3K/AKT signalling cascade, these analyses identified increased CN gains in multiple members of this pathway including: *EGFR* (*q_Fisher_* = 4.6 × 10^−3^, *q_Spearman_* = 1.7 × 10^−3^), *KRAS* (*q_Fisher_* = 1.7 × 10^−2^, *q_Spearman_* = 6.1 × 10^−3^), *PIK3R2* (*q_Fisher_* = 2.1 × 10^−2^, *q_Spearman_* = 2.4 × 10^−2^), and *AKT2* (*q_Fisher_* = 5.9 × 10^−4^, *q_Spearman_* = 2.1 × 10^−10^) (Fig. 2b). In addition to identify oncogenic genes that are known to promote HGSOC tumorigenesis, our analyses also identified a number of chromosomal regions that are frequently amplified in HGSOC, including chromosome 20q12 and 1q22 but do not contain genes that have been reported to drive tumour development or progression.

To further prioritize potential candidate oncogenes in each amplicon, we postulated that amplified oncogenic genes must also be overexpressed at the mRNA and protein levels in PPS^High^ tumours to be functionally significant. Therefore, we employed a Spearman rank correlation to identify positively (or inversely) correlated genes (Fig. 2d) and proteins (Fig. 2d) associated with poor prognosis (Table S10 and S11). By integrating these analyses, we identified 131 genes which were characterized as PPS-specific CN gains and which were overexpressed at the transcript and protein levels in PPS^High^ tumours. By assessing individual genes, we identified a number of known oncogenes including: *BCLAF1* [40], *YWHAB* [41,42], *NDRG2* [43], *NFYB* [44], *PAX8* [45,46], *PSMD14* [47]. A number of other genes that represent FDA approved drug targets including *TOP1, PARP1, HDAC2*, and *BRD4* were also included on this list of genes. Moreover, GSEA analysis [48] determined that these genes are associated with Myc or E2F activated genes, Insulin Receptor signalling, mitotic regulation, DNA replication and mRNA processing (Fig. 2f).

**Fig. 3:**
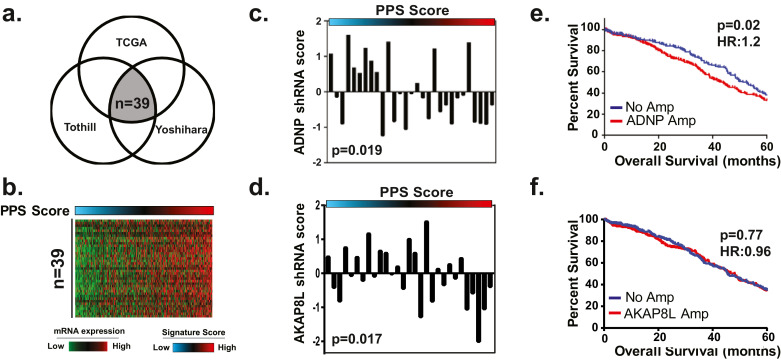
Identification of essential genes associated with PPS activity. (a) A subset of 39 genes from the 131 candidate genes were found to be consistently associated with PPS score in the TCGA, Tothill and Yoshihara datasets. (b) mRNA expression (red indicates high mRNA expression; green corresponds to low mRNA expression) of 39 candidate genes in TCGA datasets. (c-d) Analysis of data from a genome-wide RNAi screen in 29 ovarian cancer cell lines (*n* = 29) identified (c) ADNP and (d) AKAP8L as being essential for cell viability relative to the PPS score (Spearman rank correlation). (e) ADNP (*p* = 0.02; HR: 1.2) but not AKAP8L (*p* = 0.77; HR: 0.96) copy number status corresponds with overall survival in the TCGA cohort (log-rank test). (For interpretation of the references to color in this figure legend, the reader is referred to the web version of this article.)

To confirm the association between the 131 identified candidate genes and PPS, we next examined this relationship in both the Tothill [7] and Yoshihira [21] validation datasets to identify the subset of genes that were consistently associated with PPS score (Fig. 3a). Our analyses confirmed that a subset of 39 genes were consistently associated with PPS score (Fig. 3b, Figs. S2a, S2b). In order to further prioritize genes for functional analyses, we assessed the essentiality of each of the 39 genes by analysing data from a genome-wide RNA-mediated interference (RNAi) screen (∼9 k genes) performed in a panel of 29 ovarian cancer cell lines for which mRNA expression data was matched [22]. For these analyses, we compared cell line PPS score with RNAi data using a negative Spearman rank correlation to identify genes required for cell proliferation. Our analyses identified *ADNP* (*p* = 0.019) (Fig. 3c) and *AKAP8L* (Fig. 3d) (*p* = 0.017) as being amplified, overexpressed at the transcript and protein level, and essential for cell viability in context of the PPS signature. Importantly, we will note that neither *ADNP* nor *AKAP8L* are part of the PPS signature gene list. Finally, we determined that amplification of *ADNP* (*p* = 0.02 HR = 1.2) (Fig. 3e) but not *AKAP8L* (*p* = 0.77, HR = 0.96) (Fig. 3f) was prognostic in HGSOC patients, therefore *ADNP* was selected for further investigation.

### 3.3 *ADNP* is a putative oncogene associated with PPS score

**Fig. 4:**
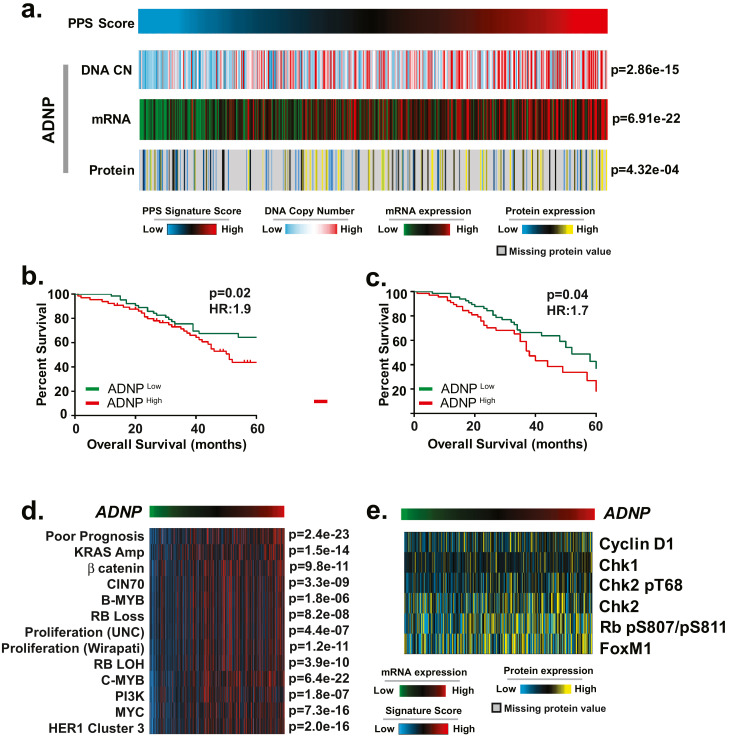
ADNP is a putative oncogene associated with PPS score. (a) ADNP copy number, mRNA, protein expression is positively correlated with PPS in the TCGA and CPTAC cohorts (FDR-corrected Spearman rank correlation). (b-c) ADNP mRNA expression (top quartile *vs* bottom quartile) corresponds with poor overall survival in the (b) Tothill cohort (*p* = 0.02; HR: 1.9) and (c) Yoshihara cohort (*p* = 0.04; HR: 1.7) by log-rank test. (d) ADNP mRNA expression (red indicates high mRNA expression; green corresponds to low mRNA expression) was found to be strongly associated with a panel of proliferation and proliferation-associated gene expression signatures in the TCGA cohort (*n* = 488) by Spearman rank correlation, (e) ADNP mRNA expression correlates with cell cycle protein expression (*p* < 0.05 for all proteins) based on RPPA analysis of 338 samples from the TCGA study by Spearman rank correlation.

As noted above, among the candidate genes associated with PPS, the transcription factor *ADNP* (Activity Dependent Neuroprotector Homeobox) was selected as being of particular interest. Our analyses identified *ADNP* DNA copy number gains (*q* = 2.8 × 10^−15^), increased mRNA expression (*p* = 6.9 × 10^−22^), and increased protein expression (*p* = 4.3 × 10^−04^) relative to PPS score (Fig. 4a). Importantly, *ADNP* mRNA expression was strongly associated with both DNA CN status (*p* = 4.1 × 10^−36^) and protein expression (*p* = 5.1 × 10^−16^) indicating that this is not a silent amplification and that *ADNP* over-expression may be functionally relevant in this subset of tumours. Consistent with this argument, analyses of clinical data in both the Tothill (Fig. 4b, *p* = 0.02; HR:1.9) and Yoshihara (Fig. 4c, *p* = 0.04; HR:1.7) datasets demonstrated that patients whose tumours have high levels of *ADNP* mRNA expression have a worse prognosis.

Given these associations, we next interrogated the relationship between *ADNP* mRNA expression and oncogenic signalling as measured by gene expression signatures (Fig. 1). As illustrated in Fig. 4d for the TGCA dataset and Fig. S3a and b for the Tothill and Yoshihara datasets, *ADNP* mRNA expression is consistently and strongly correlated with proliferation signatures (UNC, *p* = 4.4 × 10^−07^ and Wirapati, *p* = 1.2 × 10^−11^) [38,39], bMYB (*p* = 1.8 × 10^−06^) [36], cMYB (*p* = 6.4 × 10^−22^) [35] as well as RB loss (*p* = 8.2 × 10^−08^) [34], PI3K (*p* = 1.8 × 10^−7^) [25], MYC (*p* = 7.3 × 10^−16^) [25], and HER1 (*p* = 2.0 × 10^−16^) [49]. As would be expected from analysis of the TCGA samples, *ADNP* mRNA levels were strongly correlated with PPS score in the Tothill and Yoshihara datasets (Fig. S2). Further analysis of proteomic data from 338 TCGA samples demonstrated that *ADNP* mRNA levels are strongly correlated with the expression of a cell cycle proteins including Cyclin D1 (*p* = 1.2 × 10^−3^), Chk1(*p* = 2.6 × 10^−3^), Chk2(*p* = 4.7 × 10^−2^), pChk2(*p* = 7.4 × 10^−4^), pRb1(*p* = 6.5 × 10^−3^) and FoxM1(*p* = 3.8 × 10^−2^) (Fig. 4e). Collectively, these analyses suggest that *ADNP* is associated with cell cycle progression and proliferation in HGSOC tumours.

### 3.4 *ADNP* is essential for cell proliferation

Given that *ADNP* was found to be an essential gene for cancer cell line viability specifically in those cell lines with a gene expression profile associated with poor prognosis as illustrated in Fig. 3c (*p* = 0.02, *r* = −0.4) we next examined the effect of *ADNP* on tumour cell proliferation and growth in order to begin to investigate the mechanisms by which *ADNP* affects HGSOC genesis and progression. To confirm the essential role of *ADNP* in ovarian cancer proliferation, we next identified HGSOC cell lines that are characterized by high ADNP protein expression; OVCAR5 and OVCAR3 were selected for further *in vitro* experiments (Fig. S4a). We engineered OVCAR3 and OVCAR5 cell lines to express one of two tetracycline (tet)-inducible shRNA against *ADNP*. Validation studies demonstrate a consistent 60–80% reduction in *ADNP* mRNA and protein expression in OVCAR3 and OVCAR5 (Fig. S4b and c) cells following doxycycline (dox) treatment (1 µg/ml; 48 h); dox had no effect on *ADNP* expression in the parental cell line.

**Fig. 5:**
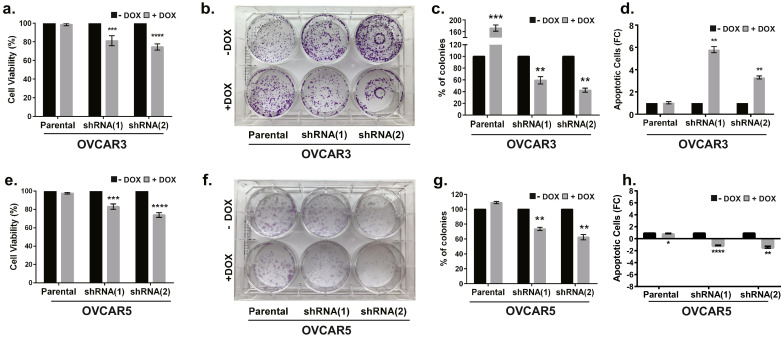
ADNP is essential for cell proliferation. (a) shRNA-mediated silencing of ADNP (48 h) resulted in a 20–30% reduction in cell viability in tet-inducible shRNA expressing OVCAR3 cell lines; no effect was noted in parent cells treated +/−dox (*t*-test). (b) Colony Formation Assay (CFA) demonstrated a significant reduction in colony formation after 14 days of shRNA-mediated silencing of ADNP in OVCAR3 cells, a modest increase in colonies was noted in the parental OVCAR3 cells treated with dox (c) Quantification of colonies (*n* = 3 replicates) demonstrated a significant 40–50% reduction in colonies in dox treated shRNA-expressing OVCAR3 cells relative to untreated cell lines; parental OVCAR3 cells show a significant increase in colony formation following dox treatment (by *t*-test). (d) 3.0–6.0 fold increase in apoptosis was observed in OVCAR3 shRNA (1) or shRNA (2) cells treated with dox, no effect was noted in parent cells treated +/-dox. (e) Similar results for cell viability were obtained in OVCAR5 cells. (f) OVCAR5 cells show decreased colony formation following *ADNP* silencing (7 days CFA). (g) Quantification of colonies (*n* = 3 replicates) demonstrate a statistically significant 40–50% decrease in colony formation in OVCAR5 cells following shRNA-mediated silencing of *ADNP*; parental cell lines show a modest, although not statistically significant increase in colony formation following dox treatment (by *t*-test). (h) 1.1- 1.4 fold decrease in apoptosis was observed in OVCAR5 shRNA (1) or shRNA (2) expressing cells +/− dox treatment (by *t*-test).

We next performed MTT and colony formation assays to determine the effect of *ADNP* on cell proliferation and survival. We determined that shRNA-mediated silencing of *ADNP* following dox treatment (1 µg/ml; 96 h) resulted in a 18.8% or 25.4% reduction in cell proliferation as measured by Cell T1itre Glow Assay in OVCAR3 cells expressing either shRNA(1) (*p* = 0.0004) or shRNA(2) (*p*<0.0001), respectively (Fig. 5a). Likewise, OVCAR5 cell lines expressing either shRNA(1) (*p*<0.0001) or shRNA(2) (*p* = 0.0005) treated with dox (1 µg/ml; 48 h) showed similar 16.9% and 26.0% reduction in cell proliferation relative to untreated control cells (Fig. 5e); neither OVCAR3 nor OVCAR5 parental cell line growth was affected by dox.

We extended these studies to assess the impact of *ADNP* silencing on long-term colony forming capabilities of these cells. While a modest increase in OVCAR3 (*p* = 0.0105) and OVCAR5 (*p* = 0.1215) parental cell colony formation was observed in response to dox treatment (1 µg/ml), we determined that OVCAR3 (Fig. 5b) or OVCAR5 (Fig. 5f) cells expressing either shRNA demonstrated a significant decrease in colony formation over a 14-day time course relative to the untreated control cells. Quantification of colonies relative to untreated control cells determined that shRNA(1) and shRNA(2) expressing OVCAR3 (Fig. 5c) cells showed a significant 40.5% (*p* = 0.0012) and 57.5% (*p* = 0.0019) reduction in colony formation relative to untreated shRNA expressing cells, respectively. Similarly, OVCAR5 cells (Fig. 5g) expressing shRNA(1) had a 26.5% reduction (*p* = 0.0032) in colonies while shRNA(2) expressing cells had a 37.5% reduction (*p* = 0.0044) in colony formation relative to untreated cells. A more significant decrease in colony formation was observed when comparing dox-treated shRNA expressing cells to the dox treated parental cells suggesting that these data may underestimate the effect of *ADNP* silencing on colony formation.

Finally, since our data indicate that *ADNP* silencing results in reduced cell growth and colony formation, we examined the impact of *ADNP* silencing on induction of apoptosis. We determined that OVCAR3 cells demonstrated a significant 5.8 fold (*p* = 0.0017) and 3.3-fold (*p* = 0.0019) increase in the combined percentage of early and late apoptotic cells following shRNA mediated silencing of ADNP for 96 h (Fig. 5d). This corresponded with an increase from 4.6% of apoptotic cells (combining early and late apoptosis) to 25.8% in shRNA(1) expressing cells (Fig. S5b and S5e) and from 5.9% to 18.8% in shRNA(2) expressing cells (Figs. S5c and S5f); no change in the percentage of apoptotic cells was observed in dox-treated parental cells (Fig. 5d, S5a and S5d). Interestingly, when these studies were repeated in OVCAR5 cells, we observed a modest decrease in apoptosis levels following *ADNP* silencing. OVCAR5 cells expressing shRNA(1) showed a 1.1-fold reduction (*p* < 0.0001) while shRNA(2) expressing cells showed 1.42-fold reduction (*p* = 0.021) (Fig. 5h and Fig. S5g–l). This corresponded with a 1.9% decrease apoptotic cells in shRNA(1) expressing cells and a 5.5% decrease in shRNA(2) expressing OVCAR5 cells. Collectively, these data indicate that ADNP is essential for cell proliferation and survival.

### 3.5 *ADNP* regulates cell cycle gene expression and *ADNP* loss induces cell cycle arrest

**Fig. 6:**
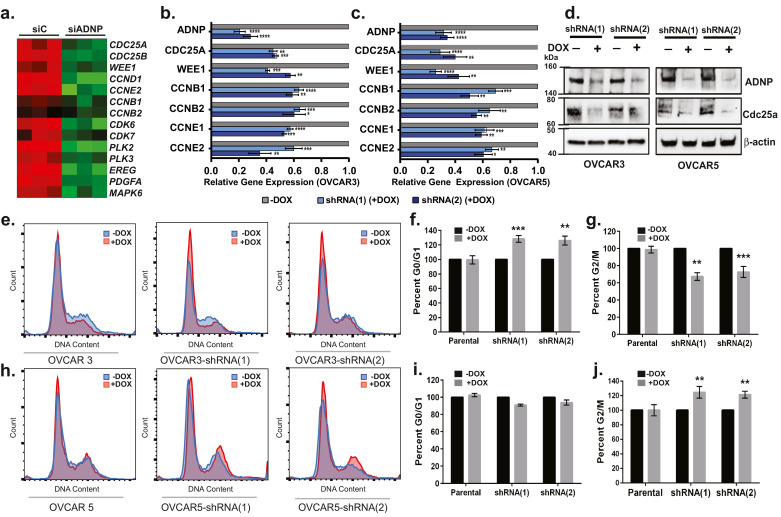
ADNP regulates cell cycle activity. (a) Multiple key cell cycle regulators including *CDC25A, CDC25B, CCNDE1, CCNB1, CCND1, CDK6*, and *WEE1* were down-regulated after siRNA-mediating silencing of ADNP (GSE79395, *n* = 6) by using FDR corrected *t*-test. (b) Treatment with 1 µg/ml doxycycline reduces ADNP mRNA expression by 70–80% as well as CDC25A mRNA expression by 60–75% and WEE1 mRNA expression by 70–80% in OVCAR3 cells expressing a tet-inducible shRNA(1) or shRNA(2) (*t*-test). (c) Similar results were observed for OVCAR5 cell lines. (d) Cdc25a protein expression is significantly reduced at the protein level following ADNP silencing in both OVCAR3 and OVCAR5 cell lines. (e) Histogram of cell cycle distribution in parental OVCAR3 and OVCAR3 cells expressing shRNA(1) or shRNA(2) +/− dox (96 h). (f) Dox-mediated ADNP silencing results in a significant increase in the percentage of OVCAR3 cells in G0/G1 and a (g) decreased in cells in G2/M (by *t*-test). (h) Histogram of cell cycle distribution in parental OVCAR5 and OVCAR5 cells expressing shRNA(1) or shRNA(2) +/− dox (96 h). (i) Dox-mediated ADNP silencing results in no change in the percentage of cells in G0/G1 but results in a (j) significant increase in cells in G2/M (by *t*-test).

Finally, analyses of publicly available gene expression data [23] demonstrated that siRNA-mediated silencing of *ADNP* results in down-regulation of 432 genes (*p* < 0.05) (Fig. S6a). Functional enrichment analysis through GSEA [48] showed that these genes play a predominant role in cell cycle checkpoints and cell cycle related pathways (Fig. S6b). As expected, further analyses of these genes identified multiple key cell cycle regulators including *CDC25A, CDC25B, CCNDE1, CCNB1, CCND1, CDK6*, and *WEE1* among others (Fig. 6a). Thus, we next validated the impact of shRNA mediated silencing of *ADNP* on the expression of a subset of these cell cycle genes in ovarian cancer cell lines. Our analyses demonstrated that shRNA-mediated silencing of *ADNP* (1 µg/ml, 96 h) resulted in a significant and reproducible reduction in *CDC25A, WEE1, CCNE1* and *CCNE2*, as well as *CCNB1* and *CCNB2* expression in OVCAR3 (Fig. 6b) and OVCAR5 (Fig. 6c) cells. Consistent with these findings, we confirmed that Cdc25a, a key regulator of both CDK2/Cyclin E activity at the G1/S transition and CDK1/Cyclin B activity at the G2/M checkpoint is significantly reduced at the protein level following *ADNP* silencing in both OVCAR3 and OVCAR5 cell lines (Fig. 6d).

Given the effect of *ADNP* on these key regulators of cell cycle progression, as well as our previous data (Fig. 4) which suggests an association between *ADNP* expression and cell cycle-related proteins and signalling pathways, we determined the impact of *ADNP* silencing on the cell cycle. As illustrated in Fig. 6e, OVCAR3 cells, including parental and shRNA(1) or shRNA(2) expressing cells, have a normal cell cycle distribution. To assess changes in the cell cycle, cells were treated with dox (1 µg/ml) for 48 h to obtain optimal silencing of *ADNP*, dox (1 µg/ml) containing medium was replaced and the effect on the cell cycle examined 48 h later. We determined that *ADNP* silencing in shRNA(1) (*p* = 0.0005) or shRNA(2) (*p* = 0.0018) expressing cells resulted in a significant arrest at the G1/S checkpoint as shown by the 28.3% and 26.0% increase in cells in G0/G1 (Fig. 6e and f). This corresponded with a similar 32.7% and 27.3% reduction of cells in the G2/M phase (Fig. 6e and g).

To confirm these observations, we examined the impact of *ADNP* silencing on the cell cycle in OVCAR5 cells. Similar to OVCAR3 cells, OVCAR5 cells showed a normal cell cycle distribution in the parental as well as shRNA(1) and shRNA(2) expressing cells (Fig. 6h). However, while silencing of *ADNP* in OVCAR5 cells had no effect on the G1/S checkpoint (Fig. 6h and i), we observed a significant 24.7% and 21.3% increase in cells in the G2/M phase in shRNA(1) (*p* = 0.0061) and shRNA(2) (*p* = 0.0014) expressing cells. These data indicate that *ADNP* is a key regulator of cell cycle genes, including *CDC25A* which has been shown to mediate both G1/S and G2/M checkpoints as well as *CCNE*, which mediates the G1/S transition and *CCNB* which is required for progression through the G2/M checkpoint.

## 4 Discussion

Although recent advances in ovarian cancer research have resulted in improved treatment strategies leading to increased overall survival of HGSOC patients, the 5-year survival rate of this disease still trails those of the vast majority of other malignancies. This highlights the need to develop approaches to identify novel, essential genes that regulate signalling networks and tumour characteristics that are required for tumour development and progression, and to develop strategies to identify the subsets of patients who will best respond to a given treatment. In this study, we undertook an integrative proteogenomic analysis of HGSOC tumours utilizing a previously published prognostic gene expression signature [3] as a conceptual framework to identify novel and essential regulators of ovarian cancer.

Our integrative *in silico* analyses of HGSOC tumour DNA copy number, mRNA and proteomic data identified *ADNP* as a potential novel driver of HGSOC tumorigenesis based on the association between poor prognosis and the expression of this gene/protein. Further analyses confirmed the prognostic capacity of *ADNP* in multiple independent datasets and suggested that *ADNP* expression is not only required for cell viability but is strongly associated with proliferation and cell cycle related signalling pathways. Indeed, *in vitro* studies confirmed the essentiality of this protein in regulating cell proliferation and survival and demonstrate a role for this gene in modulating cell cycle progression through altered expression of key cell cycle genes including *CDC25A*.

*ADNP* is a Homeobox transcription regulator which includes nine zinc-fingers that play a role in neuroprotective responses to cellular growth, chromatin remodelling, microtubule/autophagy regulation and cancer cell proliferation [9,[13], [14], [15]]. The vast majority of studies have focused on the role of *ADNP* in neurological diseases including autism spectrum disorder and Alzheimer's disease [17]. Interestingly however, it was recently reported that patients with autism-related *ADNP*-mutation syndrome, which results as a consequence of a loss of function mutation in *ADNP*, and *ADNP* haplo-insufficient mice exhibit decreased dermal thickness and wound healing due to impaired cell cycle progression [50]. More importantly, re-activation of *ADNP* in these models increased dermal thickness and activated cell cycle progression. These results are consistent with our findings that loss of *ADNP* leads to decreased expression of cell cycle genes and results in repressed cell cycle progression.

While *ADNP* has not been extensively studied in cancer, or in ovarian cancer specifically, it is localized to chromosome 20q12, a region that is frequently amplified in many malignancies including HGSOC, breast, pancreatic, and colon cancers. More recent studies from the TCGA pan-cancer project reported that while *ADNP* is rarely mutated in these tumours, it is part of a subnetwork which includes members of the SWI/SNF complex that have been shown to contribute to tumorigenesis [14]. Consistent with these observations, a number of studies have reported that *ADNP* plays a role in regulating intestinal cell growth, proliferation in specific types of sarcomas and neuronal tissue as well as modulating PI3K/ AKT signalling pathway and expression of E2F-regulated genes [15,19,24,51]. These data are consistent with our studies that show a strong correlation, in three independent datasets, between *ADNP* expression and activation of these pathways.

*ADNP* expression in HGSOC tumours is strongly associated with altered cell cycle progression and altered cell cycle checkpoints and these relationships are confirmed by analyses of down-regulated genes following ADNP silencing. Interestingly however, while shRNA-induced silencing of *ADNP* in either OVCAR3 or OVCAR5 cells results in loss of *CDC25A, WEE1, CCNE1* and *CCNE2*, and *CCNB1* and *CCNB2*, loss of *ADNP* results in G1/S checkpoint arrest in OVCAR3 cells and G2/M checkpoint arrest in OVCAR5 cells. While it is unclear why these cells arrest at different phases of the cell cycle, it is clear that loss of *ADNP* expression results in decreased *CDC25A* expression, at both the mRNA and protein levels, which has been shown to directly regulate CDK2/Cyclin E signalling at the G1/S checkpoint and CDK1/Cyclin B activity at the G2/M checkpoint [52,53]. These data would lead us to speculate that *CDC25A* activation may play a significant role in *ADNP* regulation of the cell cycle and that additional co-factors and/or cell-specific genomic alterations, including differences in p53 mutational status, may contribute to the differential response observed in OVCAR3 and OVCAR5 cells. Clearly additional studies will be required to fully delineate the mechanisms by which *ADNP* mediates the cell cycle in HGSOC.

Finally, *ADNP* mRNA expression was found to be predictive of poor overall survival in multiple independent HGSOC datasets [7,21]. While these results are contradictory to recent observations in colorectal cancer and triple negative breast cancer [23,54], which indicate that *ADNP* may play a tumour suppressive role in these cancers, they are supported by more recent studies that indicate that ADNP is oncogenic in colorectal cancer [51], pan-cancer analyses which demonstrate that ADNP is part of a SWI/SNF containing oncogenic sub-network in human cancers [44] as well as previously discussed published mechanistic studies in multiple tissue types indicating that ADNP mediates aspects of cell proliferation and growth [13,40,41,44,47]. As such our data also suggest a tissue specific or dichotomous role may exist for *ADNP* in tumorigenesis.

Collectively, our cross-platform analyses of proteogenomic data, together with *in vitro* experiments, have identified and validated *ADNP* as a novel mediator of cell proliferation in HGSOC. Although the exact mechanisms by which *ADNP* modulates ovarian cancer tumorigenesis remains to be determined, our data, in combination with previous studies, demonstrate that *ADNP* mediates its effects on HGSOC tumorigenesis, in part, by promoting dysregulation of cell cycle checkpoints. How *ADNP* abrogates this process, the identification of co-factors required for *ADNP* activity, the down-stream signalling network activated by *ADNP* in HGSOC as well as other tumour characteristics impacted by *ADNP* overexpression remain unclear. As such, defining these mechanisms will be paramount for determining the therapeutic and/or biomarker potential of *ADNP* in high-grade serous ovarian cancer as well as for other tumour types.

## Funding sources

This work was supported by R00-CA166228 from the National Cancer Institute of the National Institutes of Health and V2016-013 from the V Foundation for Cancer Research to MLG and DHFS-18PPC-024 from the New Jersey Commission for Cancer Research to KK. Rutgers Cancer Institute of New Jersey Flow Cytometry and Cell Sorting Shared Resource Facility is supported, in part, by P30-CA072720-5921. The funding organizations had no input in the design of the study; in the collection, analyses, or interpretation of the data; writing of the manuscript; or in the decision to submit the study for publication.

## Author contributions

KK and MLG conceived and designed the study. KK and MLG performed computational analyses. KK, GAM, CAK, and PK were responsible for in vitro experimental design, execution and analyses. MLG supervised all research. KK and MLG wrote the manuscript. All authors have reviewed and approved the final manuscript.

## Declaration of Competing Interests

The authors declare no competing interests.

## Appendix Supplementary materials

Image, application 1

Image, application 2

Image, application 3

Image, application 4

Image, application 5

Image, application 6

Image, application 7

Image, application 8

Image, application 9

Image, application 10

Image, application 11

Image, application 12

## References

[1] Siegel R.L., Miller K.D., Jemal A. (2019). Cancer statistics, 2019. CA Cancer J Clin.

[2] Lupia M., Angiolini F., Bertalot G., Freddi S., Sachsenmeier K.F., Chisci E. (2018). CD73 regulates stemness and epithelial-mesenchymal transition in ovarian cancer-initiating cells. Stem Cell Rep.

[3] Cancer Genome Atlas Research N (2011). Integrated genomic analyses of ovarian carcinoma. Nature.

[4] Ciriello G., Miller M.L., Aksoy B.A., Senbabaoglu Y., Schultz N., Sander C. (2013). Emerging landscape of oncogenic signatures across human cancers. Nat Genet.

[5] Matulonis U.A., Wulf G.M., Barry W.T., Birrer M., Westin S.N., Farooq S. (2017). Phase i dose escalation study of the PI3kinase pathway inhibitor BKM120 and the oral poly (ADP ribose) polymerase (PARP) inhibitor olaparib for the treatment of high-grade serous ovarian and breast cancer. Ann Oncol.

[6] Cortez A.J., Tudrej P., Kujawa K.A., Lisowska K.M. (2018). Advances in ovarian cancer therapy. Cancer Chemother Pharmacol.

[7] Tothill R.W., Tinker A.V., George J., Brown R., Fox S.B., Lade S. (2008). Novel molecular subtypes of serous and endometrioid ovarian cancer linked to clinical outcome. Clin Cancer Res.

[8] Zhang H., Liu T., Zhang Z., Payne S.H., Zhang B., McDermott J.E. (2016). Integrated proteogenomic characterization of human high-grade serous ovarian cancer. Cell.

[9] Kommoss S., Winterhoff B., Oberg A.L., Konecny G.E., Wang C., Riska S.M. (2017). Bevacizumab may differentially improve ovarian cancer outcome in patients with proliferative and mesenchymal molecular subtypes. Clin Cancer Res.

[10] Konecny G.E., Wang C., Hamidi H., Winterhoff B., Kalli K.R., Dering J. (2014). Prognostic and therapeutic relevance of molecular subtypes in high-grade serous ovarian cancer. J Natl Cancer Inst.

[11] Bowtell D.D., Bohm S., Ahmed A.A., Aspuria P.J., Bast R.C., Beral V. (2015). Rethinking ovarian cancer II: reducing mortality from high-grade serous ovarian cancer. Nat Rev Cancer.

[12] Bassan M., Zamostiano R., Davidson A., Pinhasov A., Giladi E., Perl O. (1999). Complete sequence of a novel protein containing a femtomolar-activity-dependent neuroprotective peptide. J Neurochem.

[13] Gozes I. (2018). ADNP regulates cognition: a multitasking protein. Front Neurosci.

[14] Leiserson M.D., Vandin F., Wu H.T., Dobson J.R., Eldridge J.V., Thomas J.L. (2015). Pan-cancer network analysis identifies combinations of rare somatic mutations across pathways and protein complexes. Nat Genet.

[15] Zamostiano R., Pinhasov A., Gelber E., Steingart R.A., Seroussi E., Giladi E. (2001). Cloning and characterization of the human activity-dependent neuroprotective protein. J Biol Chem.

[16] Snijders A.M., Mao J.H. (2016). Multi-omics approach to infer cancer therapeutic targets on chromosome 20q across tumor types. Adv Mod Oncol Res.

[17] Malishkevich A., Amram N., Hacohen-Kleiman G., Magen I., Giladi E., Gozes I. (2015). Activity-dependent neuroprotective protein (ADNP) exhibits striking sexual dichotomy impacting on autistic and alzheimer's pathologies. Transl Psychiatry.

[18] Mandel S., Rechavi G., Gozes I. (2007). Activity-dependent neuroprotective protein (ADNP) differentially interacts with chromatin to regulate genes essential for embryogenesis. Dev Biol.

[19] Pascual M., Guerri C. (2007). The peptide nap promotes neuronal growth and differentiation through extracellular signal-regulated protein kinase and akt pathways, and protects neurons co-cultured with astrocytes damaged by ethanol. J Neurochem.

[20] Gozes I., Yeheskel A., Pasmanik-Chor M. (2015). Activity-dependent neuroprotective protein (ADNP): a case study for highly conserved chordata-specific genes shaping the brain and mutated in cancer. J Alzheimers Dis.

[21] Yoshihara K., Tsunoda T., Shigemizu D., Fujiwara H., Hatae M., Fujiwara H. (2012). High-risk ovarian cancer based on 126-gene expression signature is uniquely characterized by downregulation of antigen presentation pathway. Clin Cancer Res.

[22] Barretina J., Caponigro G., Stransky N., Venkatesan K., Margolin A.A., Kim S. (2012). The cancer cell line encyclopedia enables predictive modelling of anticancer drug sensitivity. Nature.

[23] Blaj C., Bringmann A., Schmidt E.M., Urbischek M., Lamprecht S., Frohlich T. (2017). ADNP is a therapeutically inducible repressor of wnt signaling in colorectal cancer. Clin Cancer Res.

[24] Mermel C.H., Schumacher S.E., Hill B., Meyerson M.L., Beroukhim R., Getz G. (2011). GISTIC2.0 facilitates sensitive and confident localization of the targets of focal somatic copy-number alteration in human cancers. Genome Biol.

[25] Gatza M.L., Silva G.O., Parker J.S., Fan C., Perou C.M. (2014). An integrated genomics approach identifies drivers of proliferation in luminal-subtype human breast cancer. Nat Genet.

[26] Mehta G.A., Parker J.S., Silva G.O., Hoadley K.A., Perou C.M., Gatza M.L. (2017). Amplification of SOX4 promotes PI3K/Akt signaling in human breast cancer. Breast Cancer Res Treat.

[27] Tsherniak A., Vazquez F., Montgomery P.G., Weir B.A., Kryukov G., Cowley G.S. (2017). Defining a cancer dependency map. Cell.

[28] Bindea G., Mlecnik B., Tosolini M., Kirilovsky A., Waldner M., Obenauf A.C. (2013). Spatiotemporal dynamics of intratumoral immune cells reveal the immune landscape in human cancer. Immunity.

[29] Rody A., Holtrich U., Pusztai L., Liedtke C., Gaetje R., Ruckhaeberle E. (2009). T-cell metagene predicts a favorable prognosis in estrogen receptor-negative and HER2-positive breast cancers. Breast Cancer Res.

[30] Pfefferle A.D., Spike B.T., Wahl G.M., Perou C.M. (2015). Luminal progenitor and fetal mammary stem cell expression features predict breast tumor response to neoadjuvant chemotherapy. Breast Cancer Res Treat.

[31] Yoshihara K., Shahmoradgoli M., Martinez E., Vegesna R., Kim H., Torres-Garcia W. (2013). Inferring tumour purity and stromal and immune cell admixture from expression data. Nat Commun.

[32] Yang D., Sun Y., Hu L., Zheng H., Ji P., Pecot C.V. (2013). Integrated analyses identify a master microRNA regulatory network for the mesenchymal subtype in serous ovarian cancer. Cancer Cell.

[33] Herschkowitz J.I., He X., Fan C., Perou C.M. (2008). The functional loss of the retinoblastoma tumour suppressor is a common event in basal-like and luminal b breast carcinomas. Breast Cancer Res.

[34] Bosco E.E., Wang Y., Xu H., Zilfou J.T., Knudsen K.E., Aronow B.J. (2007). The retinoblastoma tumor suppressor modifies the therapeutic response of breast cancer. J Clin Invest.

[35] Thorner A.R., Parker J.S., Hoadley K.A., Perou C.M. (2010). Potential tumor suppressor role for the c-Myb oncogene in luminal breast cancer. PLoS ONE.

[36] Thorner A.R., Hoadley K.A., Parker J.S., Winkel S., Millikan R.C., Perou C.M. (2009). *In vitro* and *in vivo* analysis of B-Myb in basal-like breast cancer. Oncogene.

[37] Carter S.L., Eklund A.C., Kohane I.S., Harris L.N., Szallasi Z. (2006). A signature of chromosomal instability inferred from gene expression profiles predicts clinical outcome in multiple human cancers. Nat Genet.

[38] Wirapati P., Sotiriou C., Kunkel S., Farmer P., Pradervand S., Haibe-Kains B. (2008). Meta-analysis of gene expression profiles in breast cancer: toward a unified understanding of breast cancer subtyping and prognosis signatures. Breast Cancer Res.

[39] Fan C., Prat A., Parker J.S., Liu Y., Carey L.A., Troester M.A. (2011). Building prognostic models for breast cancer patients using clinical variables and hundreds of gene expression signatures. BMC Med Genom.

[40] Zhou X., Li X., Cheng Y., Wu W., Xie Z., Xi Q. (2014). BCLAF1 and its splicing regulator SRSF10 regulate the tumorigenic potential of colon cancer cells. Nat Commun.

[41] Kaneko S., Matsumoto K., Minamida S., Hirayama T., Fujita T., Kodera Y. (2016). Incremental expression of 14-3-3 protein beta/alpha in urine correlates with advanced stage and poor survival in patients with clear cell renal cell carcinoma. Asian Pac J Cancer Prev.

[42] Yuan R., Vos H.R., van Es R.M., Chen J., Burgering B.M., Westendorp B. (2018). Chk1 and 14-3-3 proteins inhibit atypical E2Fs to prevent a permanent cell cycle arrest. EMBO J.

[43] Kloten V., Schlensog M., Eschenbruch J., Gasthaus J., Tiedemann J., Mijnes J. (2016). Abundant NDRG2 expression is associated with aggressiveness and unfavorable patients' outcome in basal-like breast cancer. PLoS ONE.

[44] Fang Z., Gong C., Yu S., Zhou W., Hassan W., Li H. (2018). NFYB-induced high expression of E2F1 contributes to oxaliplatin resistance in colorectal cancer via the enhancement of CHK1 signaling. Cancer Lett.

[45] Chapel D.B., Husain A.N., Krausz T., McGregor S.M. (2017). PAX8 expression in a subset of malignant peritoneal mesotheliomas and benign mesothelium has diagnostic implications in the differential diagnosis of ovarian serous carcinoma. Am J Surg Pathol.

[46] Kar S.P., Adler E., Tyrer J., Hazelett D., Anton-Culver H., Bandera E.V. (2017). Enrichment of putative PAX8 target genes at serous epithelial ovarian cancer susceptibility loci. Br J Cancer.

[47] Zhu R., Liu Y., Zhou H., Li L., Li Y., Ding F. (2018). Deubiquitinating enzyme PSMD14 promotes tumor metastasis through stabilizing snail in human esophageal squamous cell carcinoma. Cancer Lett.

[48] Subramanian A., Tamayo P., Mootha V.K., Mukherjee S., Ebert B.L., Gillette M.A. (2005). Gene set enrichment analysis: a knowledge-based approach for interpreting genome-wide expression profiles. Proc Natl Acad Sci U S A.

[49] Hoadley K.A., Weigman V.J., Fan C., Sawyer L.R., He X., Troester M.A. (2007). EGFR associated expression profiles vary with breast tumor subtype. BMC Genom.

[50] Mollinedo P., Kapitansky O., Gonzalez-Lamuno D., Zaslavsky A., Real P., Gozes I. (2019). Cellular and animal models of skin alterations in the autism-related adnp syndrome. Sci Rep.

[51] Castorina A., Giunta S., Scuderi S., D'Agata V. (2012). Involvement of pacap/adnp signaling in the resistance to cell death in malignant peripheral nerve sheath tumor (MPNST) cells. J Mol Neurosci.

[52] Kiyokawa H., Ray D. (2008). *In vivo* roles of CDC25 phosphatases: biological insight into the anti-cancer therapeutic targets. Anticancer Agents Med Chem.

[53] Sandhu C., Donovan J., Bhattacharya N., Stampfer M., Worland P., Slingerland J. (2000). Reduction of cdc25a contributes to cyclin E1-Cdk2 inhibition at senescence in human mammary epithelial cells. Oncogene.

[54] Rangel R., Guzman-Rojas L., Kodama T., Kodama M., Newberg J.Y., Copeland N.G. (2017). Identification of new tumor suppressor genes in triple-negative breast cancer. Cancer Res.

