# Supplementary material for: Integrative proteogenomic analyses of human tumours identifies ADNP as a novel oncogenic mediator of cell cycle progression in high-grade serous ovarian cancer with poor prognosis
Source: eBioMedicine. 2019 Nov 22;50:191–202. doi: 10.1016/j.ebiom.2019.11.009 (PMC6921307; doi:10.1016/j.ebiom.2019.11.009)
Supplement: Supplementary file 1 [file mmc1.pdf]

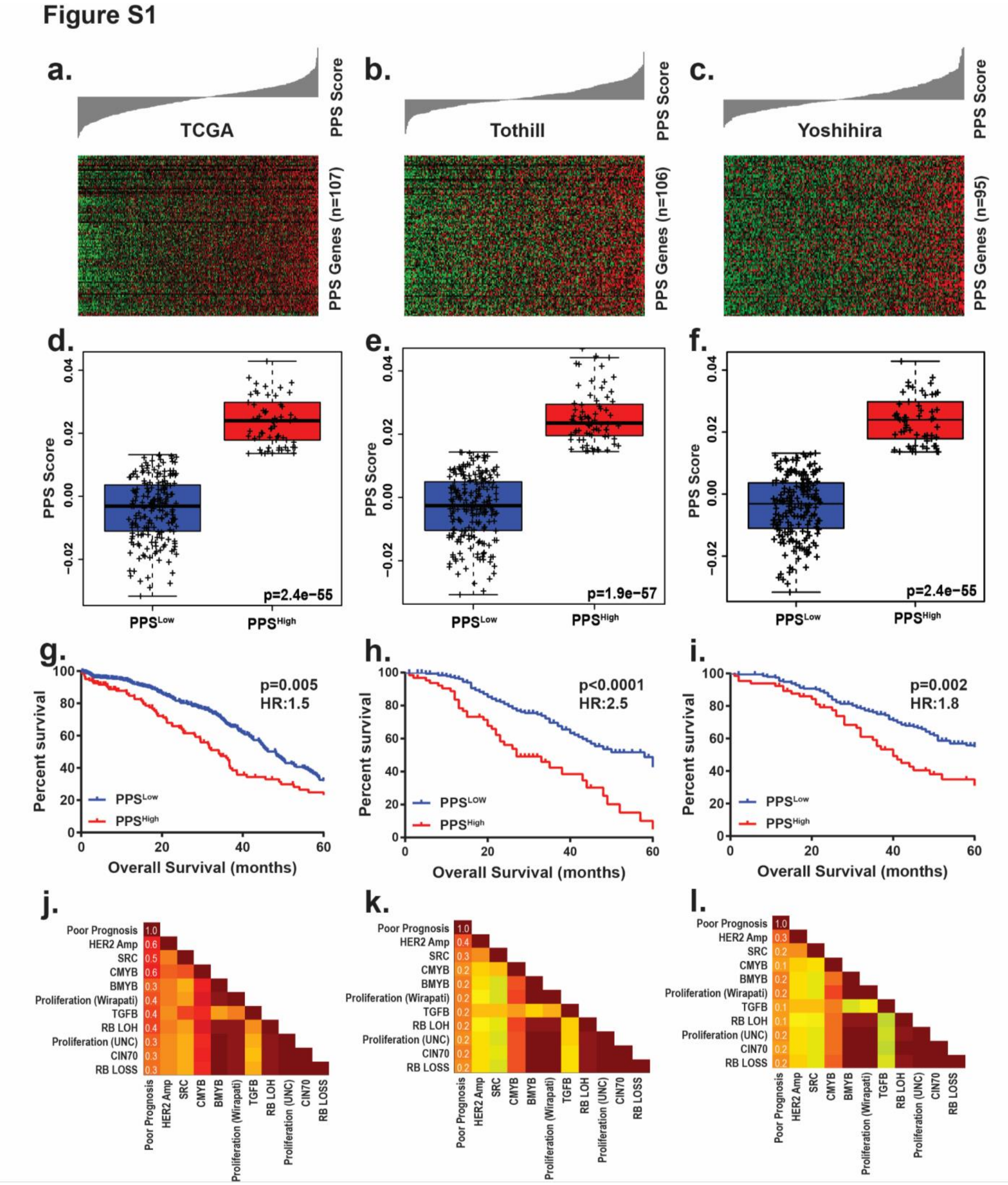

Figure S1. PPS signature is prognostic and associated with proliferation-associated signaling. PPS signature was applied to (a) TCGA, (b) Tothill and (c) Yoshihara datasets. (d-f) Samples were then divided into the top quartile (PPS^{High}) and all other samples (PPS^{Low}) in each dataset by using t-test. We confirmed the prognostic capacity of this signature in the (g) TCGA (p=0.005, HR: 1.5), (h) Tothill ($p<0.0001$, HR: 2.5), and (i) Yoshihara (p=0.002, HR: 1.8) datasets by log-rank test. PPS signature corresponds with proliferation and proliferation-associated signature in the (j) TCGA (this is the same figure as Fig. 1D shown comparison with other datasets), (k) Tothill, and (l) Yoshihara datasets. Pearson correlation coefficients for the relationship between the PPS and all other signatures are cited in each panel.

Figure S2

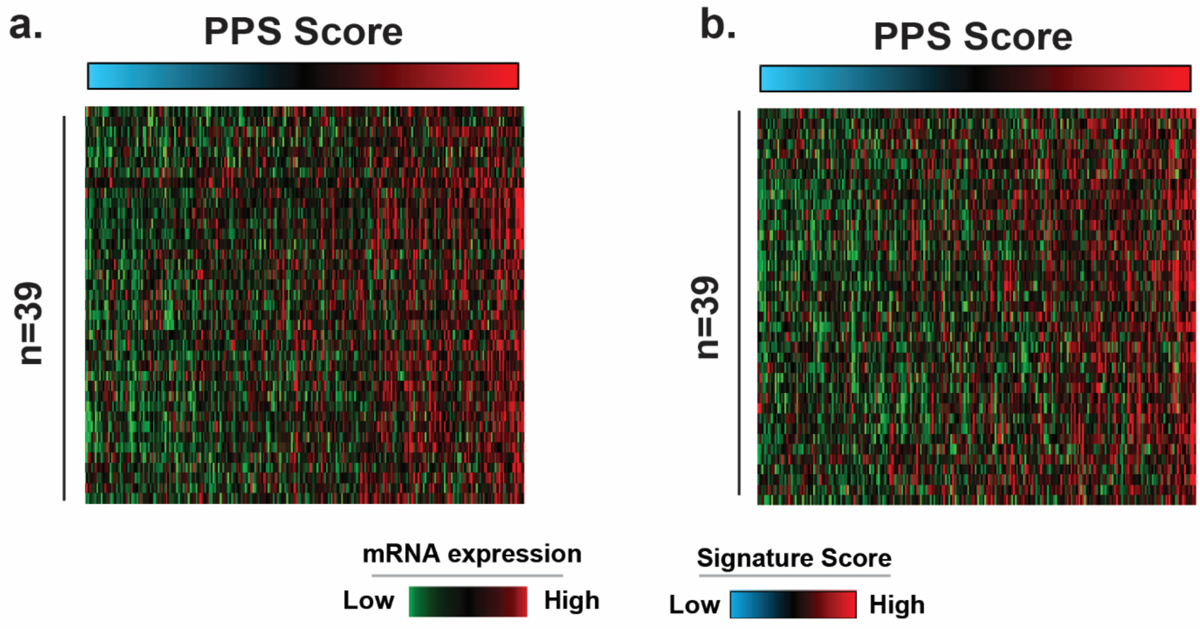

Figure 2S. mRNA expression levels of 39 genes associated with PPS activity in validation datasets. (a) Tothil and (b) Yoshihara datasets (by Spearman rank correlation).

Figure S3

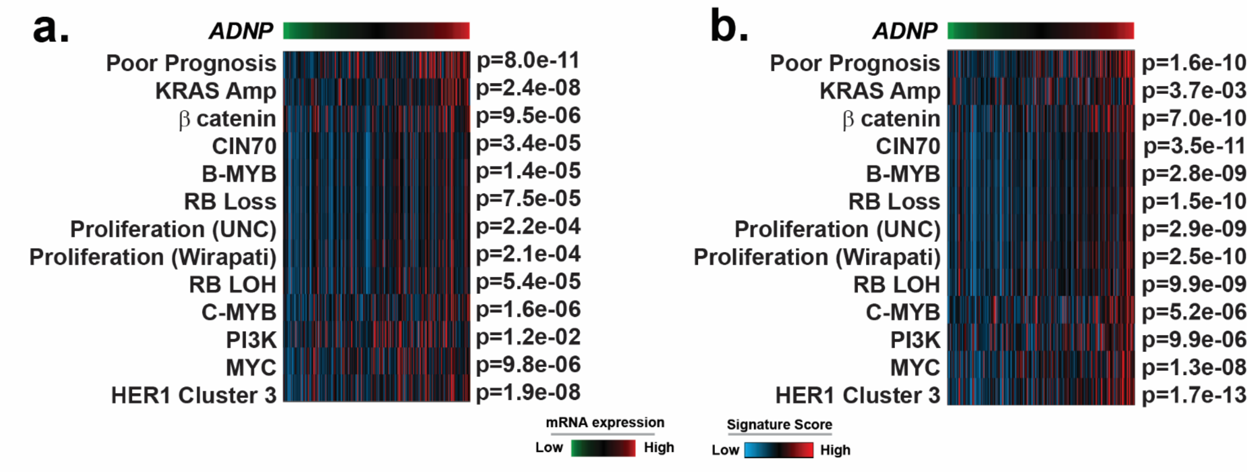

Figure S3. ADNP mRNA expression is correlated with proliferation associated signaling. ADNP mRNA expression (red indicates high mRNA expression; green corresponds to low mRNA expression) was found to be strongly associated with a panel of proliferation and proliferation-associated gene expression signatures (from Fig 1) in the (a) Tothill and (b) Yoshihara datasets (red indicates high pathway activity; blue, low pathway activity) (by Spearman rank correlation).

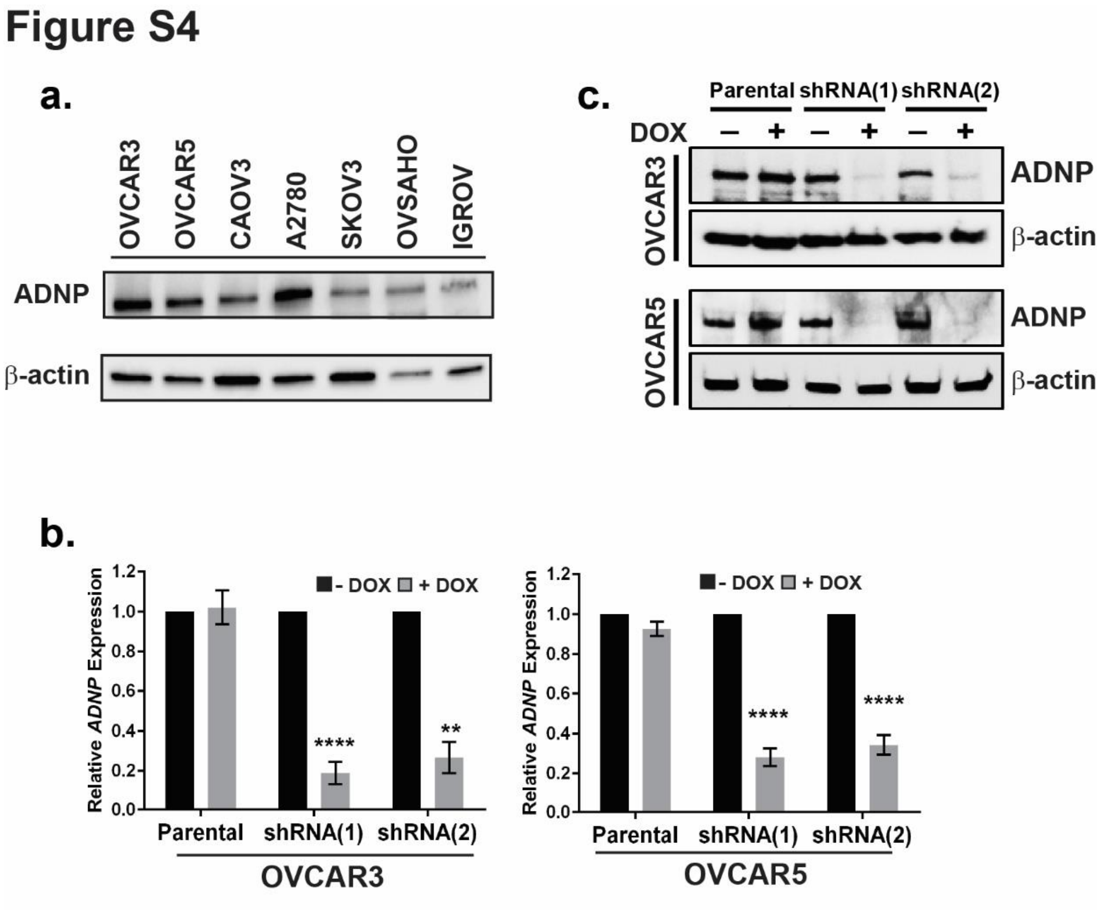

Figure S4. ADNP is essential for cell proliferation. (a) Patterns of relative ADNP protein expression in HGSOC cell line panel. (b) Treatment with 1μg/ml doxycycline reduces ADNP mRNA expression by 70-80% in OVCAR3 cells expressing a tet-inducible shRNA(1) or shRNA(2); no effect was observed in parental OVCAR3 cell lines. Similar results were observed for OVCAR5 cell lines (by t-test). (c) ADNP protein levels were similarly reduced following dox treatment of shRNA(1) or shRNA(2) expressing OVCAR3 and OVCAR5 cells; no effect on ADNP expression was observed in western blot analyses of parental cell lines +/- dox.

Figure S5

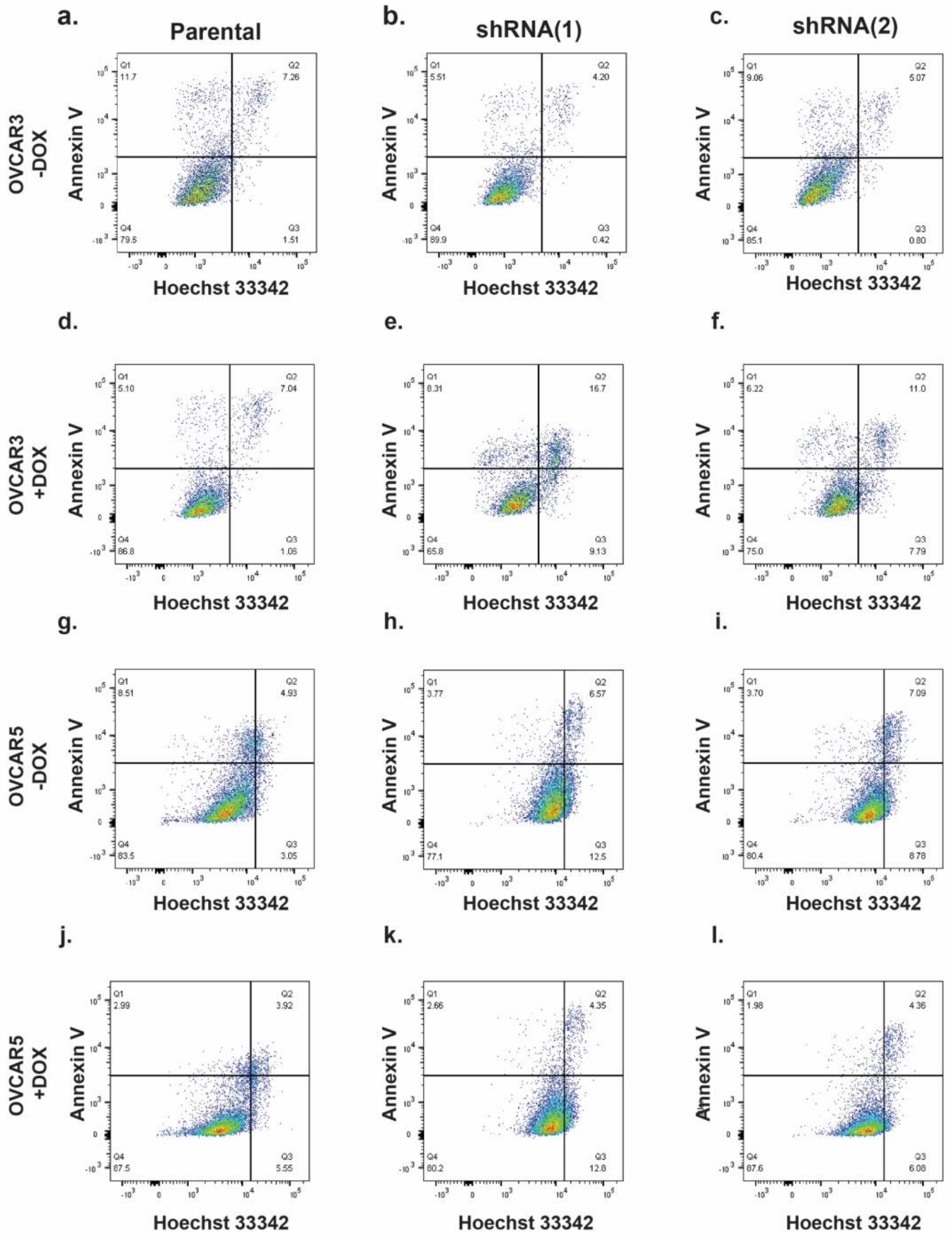

Figure S5. *ADNP* silencing impacts on induction of apoptosis. FACS analysis was used to determine the frequency of apoptotic cells in OVCAR3 and OVCAR5 cells expressing either shRNA(1) or shRNA(2) or parental cells. Representative FACS scatter plots are shown here. (a) Parental OVCAR3 cells, (b) OVCAR3 shRNA(1) and (c) OVCAR shRNA(2) expressing cells show no increase in apoptic cells in the absense of dox treatment. (d) Dox treatment has no effect on the percent of apoptotic cells in parental OVCAR3 cells. (e) ShRNA-mediated silencing of ADNP over 96 hours resulted in an increased from 4.6% of apoptotic cells to 25.8% in shRNA(1) expressing cells in OVCAR3 cell line and (f) an increase from 5.9% to 18.8% in shRNA(2) expressing cells. (g-i) OVCAR5 parental, shRNA(1) or shRNA(2) expressing cells show no difference in the percent of apopotic cells. (j) Dox treatment of parental OVCAR5 cells results in a modest increase in apoptosis. (k) ShRNA-mediated silencing of ADNP (96 hours) resulted in a decrease from 19.1% of apoptotic cells to 17.1% in OVCAR5 shRNA(1) expressing cells and (l) a decrease from 15.9% to 10.4% in shRNA(2) expressing cells in OVCAR5 cell lines. Hoechst33342 DNA staining is quantified on the x-axis and Annexin V –FITC expression is reported on the y-axis of each graph.

Figure S6

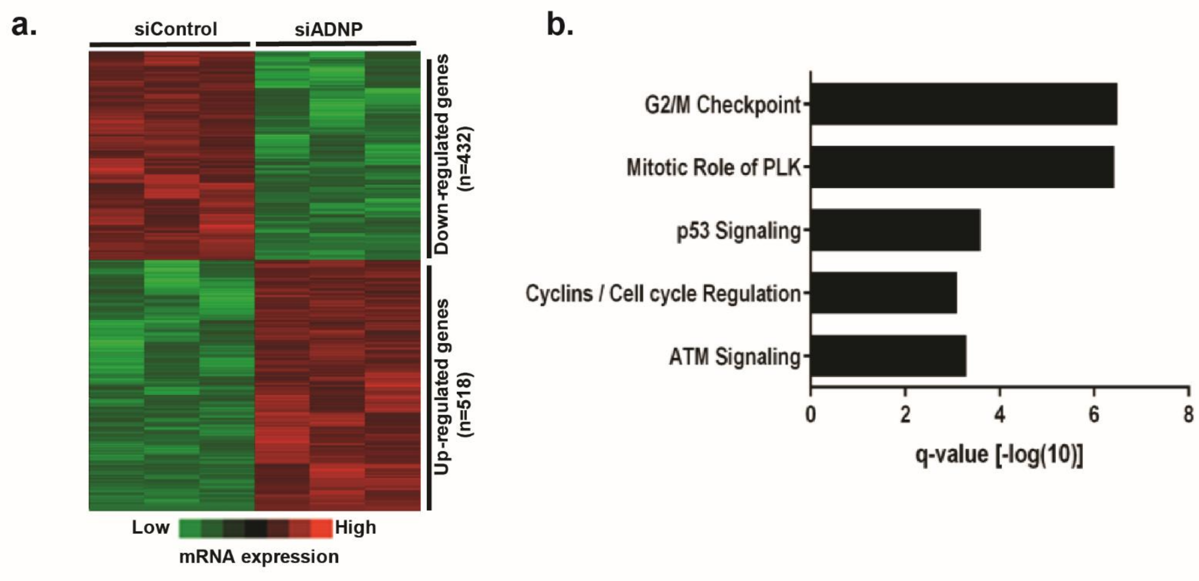

Figure S6. ADNP regulates cell cycle activity. (a) siRNA-mediated silencing of ADNP (GSE79395, n=6) resulted in decreased expression of 432 genes and increased expression of 518 genes (t-test-FDR corrected, q<0.05). (b) Functional enrichment analysis demonstrated that down-regulated genes mediate multiple cellular processes including cell cycle and related pathways.
